# Rectal syphilis presenting as a bleeding rectal tumour

**DOI:** 10.1093/jscr/rjag425

**Published:** 2026-06-03

**Authors:** Kenneth Thorsen, Lars Peter Christersson, Jacob Jose Fortes Goldman, Ole Jacob Greve, Dordi Lea

**Affiliations:** Department of Gastrointestinal Surgery, Stavanger University Hospital, Leif Larsens gate 8, 4021 Stavanger, Norway; Section for Traumatology, Stavanger University Hospital, Leif Larsens gate 8, 4021 Stavanger, Norway; Department of Clinical Medicine, University of Bergen, Postbox 7804, 5020 Bergen, Norway; Stavanger Medical Centre, Sverdrups gate 23, 4007 Stavanger, Norway; Department of Dermatology and Venereal Diseases, Stavanger University Hospital, Leif Larsens gate 8, 4021 Stavanger, Norway; Department of Radiology, Stavanger University Hospital, Leif Larsens gate 8, 4021 Stavanger, Norway; Department of Pathology, Stavanger University Hospital, Leif Larsens gate 8, 4021 Stavanger, Norway

**Keywords:** rectal bleeding, rectal cancer, low anterior resection syndrome, syphilis

## Abstract

Rectal bleeding is a very common symptom referred to hospitals for proctoscopy daily. We present a rare case of a bleeding mass in the rectum. A male patient in his 40s was referred to hospital due to rectal bleeding. On the digital rectal examination, a polypus rectal mass was palpated. Biopsies were taken, and the magnetic resonance imaging (MRI) described a 5 cm long tumour. Multiple enlarged lymph nodes in the area were seen on MRI also outside the mesorectum with possible venous infiltration. Radiologically, it was categorized as T3bN2. Due to the patient history, a test for syphilis was requested, which confirmed the diagnosis. Hence, the rectal tumour was identified as a primary chancre of syphilis. Rectal syphilis is a rare manifestation of syphilis, but must be kept in mind as a differential diagnosis when encountering a bleeding rectal mass, especially in men having sex with men.

## Introduction

Rectal bleeding is a very common symptom referred to hospitals for proctoscopy daily.

The vast majority of patients present with haemorrhoids, often accompanied by different grades of constipation, but premalignant or malignant lesions are not uncommon, even in younger age groups [1]. Therefore, even when haemorrhoids are found, clinicians should always evaluate whether the bleeding may be caused by a tumour in the lower gastrointestinal tract. If the bleeding is fresh and caused by a tumour, it is usually located in the rectum or lower colon. In the early stages of colorectal cancer (CRC), there may not be symptoms other than bleeding, with no signs of weight loss, anaemia, fatigue, or constipation. However, when such symptoms are present, CRC is more likely.

Syphilis is caused by the spirochete *Treponema pallidum* and is a sexually transmitted disease [2]. The disease is known as the great mimicker, because its symptoms can mimic a wide range of diseases [2]; however, in high-income countries, it has been a rarity in modern years. Nonetheless, the incidence has been rising even in high-income countries among men having sex with men [3, 4]. Rectal syphilis has previously been described in case reports, where a bleeding rectal mass can be mistaken for a malignant rectal tumour [5–10]. However, this scenario is rare, and it is important to be aware of to initiate proper treatment and avoid causing harm.

We present such a case referred to our institution.

## Case report

A male patient in his 40s was referred by his general practitioner (GP) to hospital due to rectal bleeding. The patient had known haemochromatosis and hyperlipidaemia but was otherwise healthy. He had contacted his GP due to constipation, fatigue, weight loss, and perianal itching. The patient was under treatment with semaglutide, which could account for the weight loss, constipation, and fatigue. He also complained of pain in the right hip and in both groynes and had been suffering from poor sleep and increased sweating. On initial examination at the GP’s office, a large skin tag was found in the perianal area, but there was no evidence of haemorrhoids. On his way out from the GP’s office, the patient mentioned that a previous partner had tested positive for syphilis, and hence a serological test for syphilis was performed. The result came back inconclusive, and a new test was recommended after 2–3 weeks.

On the digital rectal examination (DRE) at the hospital, a polypus rectal mass was palpated ventrally about 8 cm in from the anal verge. The mass was not typical of cancer, and there were several ulcers nearby the tumour. However, due to the findings on the DRE and proctoscopy, the mass was still macroscopically suspicious of cancer, and several biopsies were taken, and a magnetic resonance imaging (MRI) was performed.

**Figure 1:**
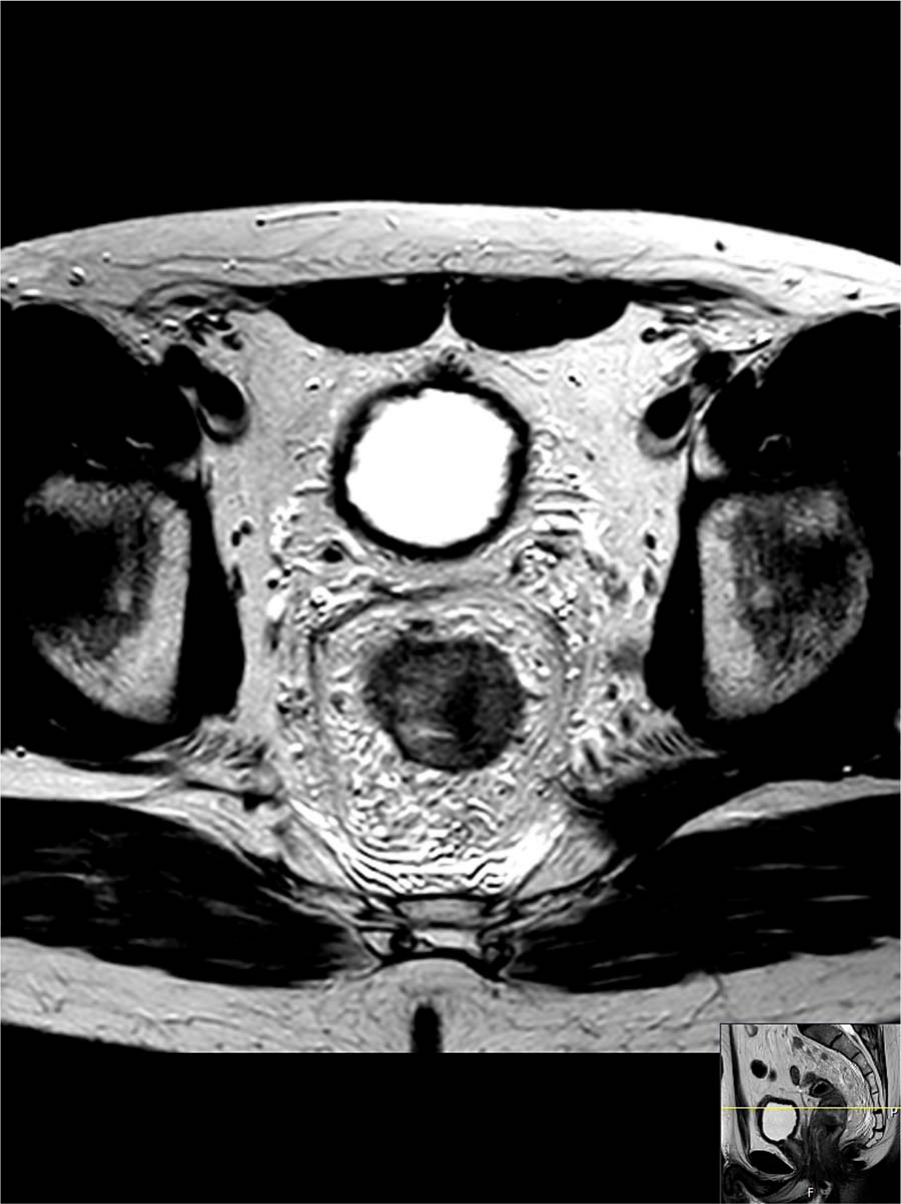
A cross-section of the MRI shows a rectal tumour mass located between 4 and 8 o’clock; the shortest distance to the mesorectal fascia was 4 mm from the tumour and 1 mm from a lymph node that appears malignant; there were multiple enlarged lymph nodes in the area, also outside the mesorectum with possible venous infiltration; radiologically categorized as T3bN2.

The MRI described a 5 cm long tumour located between 4 and 8 o’clock. There was an impression of oedema and inflammation in the mesorectal fat and surrounding lymph nodes. Multiple enlarged lymph nodes in the area were seen on the MRI also outside the mesorectum with possible venous infiltration. Radiologically, the lesion was categorized as T3bN2 (Fig. 1).

The blood tests showed a normal glycated haemoglobin (HbA1c) of 34 mmol/L. Erythrocyte sedimentation rate (ESR) was elevated to 23 mm/h, and C-reactive protein (CRP) was slightly elevated to 17.6 mg/L. While thyroid-stimulating hormone (TSH) was normal at 2.1 ml/U, free thyroxine (FT4) was decreased to 8.2 pmol/L. Ferritin was elevated to 1032 μg/L, and alanin aminotransferase (ALT) was elevated to 150 U/L, though gamma glutamyltransferase (GGT) remained normal at 56 U/L.

Several other tests were inconspicuous, and the HIV-test was negative.

The patient took a new blood test after 2 weeks. At this time, he also contacted the GP by e-consultation because of a red maculopapular rash affecting several parts of the body. The second syphilis test came back positive for an active syphilis infection, and the patient was referred to the dermatology and venereology department.

**Figure 2:**
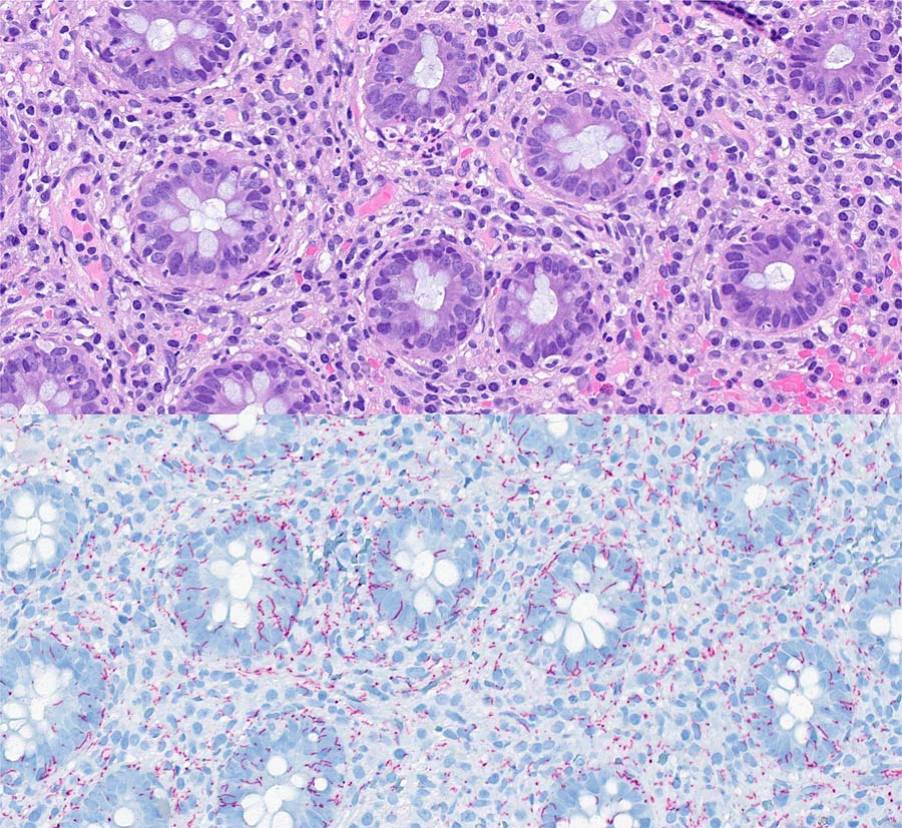
Histopathological features of the biopsy specimen from rectal syphilis showing a well-preserved epithelium with some inflammation and no dysplasia (a—depicted in purple); in the lamina propria, there is a diffuse lymphoplasmacytic infiltrate (haematoxylin and eosin stain ×4000); immunohistochemical staining for *Treponema pallidum* (b—depicted in blue) verifies a large number of spirochetes (×4000).

The biopsies showed inflammation. No stigma of malignancy was identified. After the information from the GP about active syphilis infection came to the hospital, the pathologist was consulted and asked for the possibility to check for *T. pallidum*. Due to a lack of immunohistochemical staining for *T. pallidum* at our institution, the sample was sent to another hospital where the diagnosis was confirmed (Fig. 2). Hence, the rectal tumour was identified as a primary chancre of syphilis.

**Figure 3:**
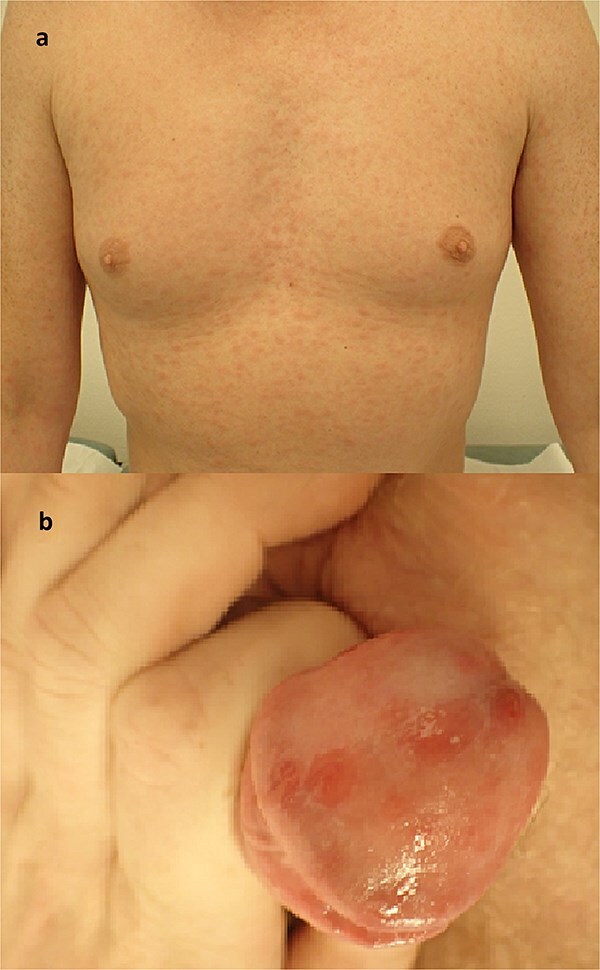
Photos taken at the outpatient clinic for dermatology and venereal disease showing a non-itchy, generalized red maculopapular rash of the chest and arms (a); the same rash affected several parts of the body; in addition, the patient had penile sores, which were not the primary chancre or site of inoculation, but rather a cutaneous presentation of the systemic affliction of the secondary stage of the disease (b).

The dermatologist identified several skin lesions commonly seen in cutaneous syphilis, including a typical penile lesion depicted in Fig. 3. These skin lesions were not present at the initial contact with the GP and hospital. Antibiotics were started, and 6 weeks later, the patient was feeling considerably better.

## Discussion

A primary chancre mimicking a malignant rectal tumour is rare, but has been described in other case reports [5–8]. The consequences of a mistaken diagnosis may be enormous.

The inoculation period for syphilis is 10–70 days, during which the primary chancre usually is a painless ulcer found at the inoculation site. This may be encountered in any mucosal ulcer or erosion [4]. In this specific case, the MRI indicated a locally advanced tumour, where preoperative radiation would have been the preferred treatment due to lymph node involvement [11]. The tumour was located 8 cm from the anal verge, and a low anterior resection with a colorectal anastomosis would have been achievable, but a lower tumour would have led to a formal rectal amputation with a permanent end colostomy. In addition, the side effects of pelvic radiation may include a bothersome skin lesion, proctitis, enteritis, and a higher risk of anastomotic leakage and low anterior resection syndrome. Thus, a thorough patient history and proper biopsy sampling are of utmost importance. If the syphilis diagnosis had not reached the hospital, there is a possibility that *T. pallidum* would not have been tested for. The clinical findings together with the MRI findings could, in the lack of a positive identification of *T. pallidum*, have led to unnecessary treatment with preoperative radiation and a low anterior resection for this particular patient.

Rectal syphilis is a rare manifestation of syphilis but must be kept in mind as a differential diagnosis when encountering a bleeding rectal mass, especially in men having sex with men.

## References

[1] Colles T, Ziegelmann PK, Damin DC. The role of colonoscopy in young patients with rectal bleeding: a systematic review and meta-analysis. Int J Color Dis 2023;38:230. 10.1007/s00384-023-04524-4 37712988

[2] Peeling RW, Mabey D, Kamb ML et al. Syphilis. Nat Rev Dis Primers 2017;3:17073. 29022569 10.1038/nrdp.2017.73 PMC5809176

[3] Janier M, Unemo M, Dupin N et al. 2020 European guideline on the management of syphilis. J Eur Acad Dermatol Venereol 2021;35:574–88. 33094521 10.1111/jdv.16946

[4] Peeling RW, Mabey D, Chen XS et al. Syphilis. Lancet 2023;402:336–46. 37481272 10.1016/S0140-6736(22)02348-0

[5] Zhao WT, Liu J, Li YY. Syphilitic proctitis mimicking rectal cancer: a case report. World J Gastrointest Pathophysiol 2010;1:112–4. 21607150 10.4291/wjgp.v1.i3.112 PMC3097953

[6] Febbraro I, Manetti G, Balestrieri P et al. Rectal cancer or rectal chancre? Beware of primary syphilis. Dig Liver Dis 2008;40:579–81. 18313998 10.1016/j.dld.2007.09.004

[7] Costales-Cantrell JK, Dong EY, Wu BU et al. Syphilitic proctitis presenting as a rectal mass: a case report and review of the literature. J Gen Intern Med 2021;36:1098–101. 33469766 10.1007/s11606-020-06414-9 PMC8042098

[8] Peine B, Ved KJ, Fleming T et al. Syphilitic proctitis presenting as locally advanced rectal cancer: a case report. Int J Surg Case Rep 2023;107:108358. 37267792 10.1016/j.ijscr.2023.108358 PMC10310909

[9] Tigchelaar CK, Brosens LAA, Seldenrijk CA. An unusual rectal mass. Gastroenterology 2012;143:e16–7. 22841728 10.1053/j.gastro.2012.02.047

[10] Cha JM, Choi SI, Lee JI. Rectal syphilis mimicking rectal cancer. Yonsei Med J 2010;51:276–8. 20191023 10.3349/ymj.2010.51.2.276 PMC2824876

[11] Noticewala SS, Das P. Current state of neoadjuvant therapy for locally advanced rectal cancer. Cancer J 2024;30:227–31. 39042772 10.1097/PPO.0000000000000725

